# Diabetes Mellitus and Endo-Periodontal Lesions: An HbA1c-Guided Framework for Clinical Decision-Making

**DOI:** 10.3390/medicina62071420

**Published:** 2026-07-22

**Authors:** Adrian Stan, Mihaela Moisei, Liliana-Lăcrămioara Pavel, Liliana Mititelu-Tarțău, Beatrice Rozalina Buca, Mioara Decusară

**Affiliations:** 1 Faculty of Medicine and Pharmacy, Medical and Pharmaceutical Research Center, Dunarea de Jos University, 800008 Galati, Romania; adrian.stan@ugal.ro (A.S.); liliana.pavel@ugal.ro (L.-L.P.); mioara.decusara@ugal.ro (M.D.); 2 Grigore T. Popa University of Medicine and Pharmacy Iasi, 700115 Iasi, Romania; lylytartau@yahoo.com (L.M.-T.); beatriceroza@yahoo.com (B.R.B.)

**Keywords:** endo-periodontal lesions, diabetes mellitus, HbA1c, apical periodontitis, periodontitis, glycemic control, clinical decision-making, oral-systemic health

## Abstract

*Background and Objectives*: Diabetes mellitus is a major systemic modifier of oral infection, periodontal inflammation, and tissue healing. Persistent hyperglycemia may intensify oxidative stress, immune dysfunction, dysbiotic biofilm activity, and advanced glycation end-product/receptor signaling, thereby reducing the predictability of periapical and periodontal healing. In patients with endo-periodontal lesions, these mechanisms may interact with pulpal infection and periodontal breakdown, complicating diagnosis, prognosis, and therapeutic sequencing. This review summarizes current evidence on diabetes, glycated hemoglobin (HbA1c), and endo-periodontal outcomes and proposes an HbA1c-guided clinical framework for risk stratification and treatment planning. *Materials and Methods*: A structured narrative review with an expert-informed clinical framework was conducted using the literature published between January 2016 and December 2025. Eligible sources included consensus reports, clinical practice guidelines, systematic reviews, meta-analyses, umbrella reviews, randomized and non-randomized clinical studies, observational studies, and selected mechanistic studies or contemporary narrative reviews with direct relevance to the framework. Case reports, animal studies, and in vitro investigations without direct relevance to the clinical framework were excluded from the main synthesis. *Results*: Diabetes is associated with increased periodontal severity, impaired periodontal treatment response, a higher prevalence of radiolucent periapical lesions in root-filled teeth, delayed periapical healing, and increased non-retention of endodontically treated teeth. Periodontal therapy is safe in diabetic patients and may be associated with modest reductions in HbA1c, especially when baseline metabolic control is poor. *Conclusions*: HbA1c should complement, not replace, pulpal testing, periodontal charting, and radiographic assessment. The proposed framework prioritizes endodontic infection control when pulpal necrosis or active intracanal infection is present, adapts periodontal intervention to metabolic risk, and postpones elective surgical or regenerative procedures in poorly controlled diabetes until medical stabilization is achieved.

## 1. Introduction

Evidence-mapping approaches help organize heterogeneous clinical information when the available literature combines methodological guidance, consensus statements, observational evidence, clinical trials and review-level data [1]. Endo-periodontal lesions are clinical entities in which the dental pulp and periodontal supporting tissues communicate through inflammatory, anatomical and microbial pathways. The apical foramen, lateral canals, accessory canals, dentinal tubules and iatrogenic or traumatic root defects create routes that can connect the root canal system with the periodontal compartment. These pathways explain why pulpal necrosis may present with periodontal drainage and why advanced periodontal destruction may complicate pulpal diagnosis [2,3].

The 2017 World Workshop classification placed endo-periodontal lesions among clinically relevant periodontal conditions and separated lesions with root damage from lesions without root damage. This distinction has direct therapeutic consequences because vertical root fracture, perforation or external root resorption frequently changes prognosis and may contraindicate regenerative periodontal treatment [4,5,6]. Recent expert consensus also emphasizes that diagnosis should integrate pulpal status, periodontal probing pattern, radiographic morphology, presence of sinus tract, tooth restorability and patient-related risk factors [2]. Microbial interactions between root canals and periodontal pockets further support the need for an integrated diagnostic approach [7].

Diabetes mellitus represents one of the most important systemic modifiers of periodontal inflammation and tissue healing. Hyperglycemia alters host defense, increases oxidative stress, promotes accumulation of advanced glycation end products, activates receptor-mediated inflammatory signaling and interferes with collagen metabolism and bone repair [8,9,10,11,12]. These mechanisms affect both marginal periodontal tissues and periapical tissues, so diabetic status is highly relevant when the clinician evaluates teeth with combined endodontic and periodontal pathology.

Glycated hemoglobin (HbA1c) provides a practical estimate of glycemic exposure over the preceding 8 to 12 weeks. In dental medicine, HbA1c has clinical value because it reflects metabolic conditions that influence infection control, inflammatory activity, wound healing and risk of postoperative complications. Periodontal treatment in diabetic patients may produce modest short-term improvements in glycemic control, particularly when baseline HbA1c is elevated [13,14,15,16,17]. In periodontology, the staging and grading framework recognizes uncontrolled diabetes as a grade modifier and supports the incorporation of systemic risk factors into diagnosis and planning [5]. In endodontics, systematic reviews, meta-analyses and clinical evidence have reported an association between diabetes and higher prevalence of periapical radiolucencies, less favorable periapical healing, increased non-retention of root-filled teeth and altered outcomes in patients with apical periodontitis [18,19,20,21,22,23,24].

Despite these converging data, there is no universally accepted clinical guideline that specifically stratifies endo-periodontal lesion management according to HbA1c levels. Existing recommendations address periodontitis, diabetes, root canal treatment outcomes or endo-periodontal diagnosis separately. The clinician therefore needs a structured framework that combines local diagnosis with systemic metabolic status without overinterpreting HbA1c as a stand-alone diagnostic test.

**Figure 1:**
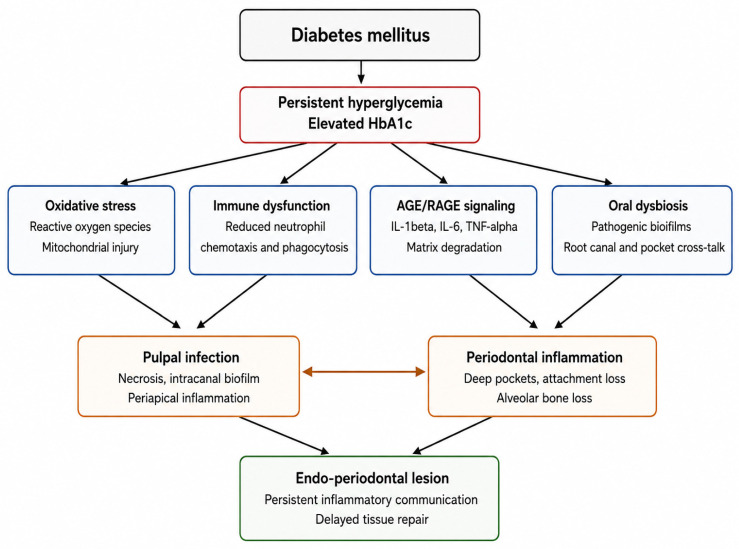
Pathobiological links between diabetes mellitus, persistent hyperglycemia, periodontal inflammation and endo-periodontal lesion progression.

The objective of this review is to map recent evidence on diabetes, HbA1c, periodontal inflammation, periapical disease and endo-periodontal management, and to translate these findings into an HbA1c-guided clinical practice framework. The framework aims to support diagnostic accuracy, therapeutic sequencing, surgical timing, risk communication and supportive periodontal and endodontic maintenance. The main pathobiological connections underlying this framework are summarized in Figure 1.

## 2. Materials and Methods

### 2.1. Review Design

This study was conducted as a structured narrative review with an expert-informed clinical framework. The methodological structure was informed by current guidance for evidence mapping and structured review methodology [1]. The manuscript was not designed as a full systematic review or meta-analysis. Instead, it was intended to integrate heterogeneous evidence from different levels of the clinical literature and translate it into a practical decision-making model.

Pooled effect sizes were not calculated because the available evidence included consensus reports, clinical guidelines, observational studies, clinical trials, systematic reviews and meta-analyses with heterogeneous populations, interventions, follow-up periods and outcome measures. The synthesis therefore aimed to organize the available evidence into clinically relevant domains, identify variables with prognostic and therapeutic relevance, and develop a structured risk-oriented framework for dental practice.

The clinical framework was developed through thematic integration of the selected evidence and was structured around four decision domains: pulpal diagnosis, periodontal diagnosis, metabolic status and treatment sequencing. These domains were considered together to support individualized prognosis, selection of treatment intensity and determination of appropriate follow-up. HbA1c was treated as a prognostic and planning variable, not as a diagnostic substitute for pulp vitality testing, periodontal charting or radiographic assessment.

### 2.2. Research Question and PCC Framework

The research question was: In diabetic patients with endo-periodontal lesions or overlapping endodontic and periodontal inflammatory disease, how can HbA1c and glycemic control inform diagnosis, prognosis and clinical management? The question was structured using the Population–Concept–Context framework to ensure a clear connection between the target patient group, the main clinical variables and their application in dental practice. This approach supported the organization of the evidence around metabolic risk, local diagnosis, treatment sequencing and follow-up. The Population–Concept–Context framework is presented in Table 1.

### 2.3. Search Strategy and Source Selection

The search strategy was developed for PubMed, Scopus and Web of Science and covered the literature published between January 2016 and December 2025. The core search combined terms related to diabetes mellitus, hyperglycemia and HbA1c with concepts referring to periodontal disease, apical periodontitis, endodontic treatment and endo-periodontal lesions. Search terms were combined using Boolean operators and adapted to the indexing system and syntax of each database. This approach was intended to identify evidence relevant to the interaction between metabolic control, local inflammatory disease, healing response and clinical treatment outcomes.

The core database search was complemented by targeted searches focused on intracanal medicaments, calcium silicate-based bioceramic materials, and local periodontal drug-delivery systems. These additional searches were undertaken to ensure adequate coverage of contemporary therapeutic options relevant to endodontic disinfection and adjunctive periodontal inflammation control in patients with diabetes.

Reference lists of major consensus reports, clinical practice guidelines, systematic reviews and meta-analyses were also screened to identify additional relevant publications that might not have been retrieved through the initial search combinations. Priority was given to sources with clear methodological reporting, direct clinical applicability and relevance to diagnosis, prognosis, treatment sequencing or follow-up. The database-specific core and targeted search strings, together with the applied limits, are presented in Table 2.

### 2.4. Eligibility Criteria

Sources were considered eligible when they addressed at least one of the following domains: diabetes and periodontitis, diabetes and apical periodontitis, HbA1c and oral inflammatory outcomes, diagnosis and prognosis of endo-periodontal lesions, therapeutic sequencing, periodontal treatment in patients with diabetes, endodontic outcomes in diabetic patients, or regenerative and surgical management of combined lesions. Studies were included when their findings contributed directly to the clinical interpretation of metabolic risk, local disease activity, healing potential, treatment timing or follow-up planning.

Priority was given to systematic reviews, meta-analyses, umbrella reviews, consensus reports, clinical practice guidelines, randomized clinical trials, cohort studies and clinically relevant retrospective investigations. Sources with transparent methodology, clearly defined populations, clinically interpretable outcomes and direct relevance to the proposed HbA1c-guided framework were preferentially retained. Contemporary narrative reviews were considered when they provided a clinically relevant synthesis of mechanisms or therapeutic materials not fully addressed by higher-level evidence.

Case reports and purely educational clinical scenarios were not included in the main evidence synthesis because of their limited generalizability. Animal studies and in vitro investigations were excluded when direct clinical extrapolation was not possible. Articles not published in English, conference abstracts without full-text availability and sources lacking clearly defined clinical or biological outcomes were also excluded.

Selected mechanistic in vitro studies were retained only when they directly addressed the biological properties of contemporary endodontic materials relevant to the clinical framework. These studies were used exclusively to support mechanistic interpretation, including biocompatibility, inflammatory modulation, mineralization and osteogenic potential, and were not used to establish comparative clinical superiority or definitive treatment recommendations.

The eligibility criteria used for source selection and evidence mapping are summarized in Table 3.

### 2.5. Evidence Charting and Source Classification

The included sources were classified according to their direct relevance to the structured narrative review and the HbA1c-guided clinical framework. The methodological literature addressing evidence mapping and structured review reporting was retained to support the organization of the manuscript [1]. Clinical sources were assessed according to thematic relevance, DOI traceability, publication type, clinical applicability and contribution to the proposed decision-making framework.

For each clinical or biological source, the following information was charted: author, year, study type, population or clinical context, diabetes definition, HbA1c reporting, periodontal diagnosis, endodontic diagnosis, intervention, follow-up, numerical outcomes and clinical implications. The included clinical and biological sources were grouped into four evidence categories: guidelines, consensus reports and position statements; systematic reviews, meta-analyses and umbrella reviews; clinical, interventional and observational studies; and mechanistic studies or contemporary narrative reviews.

To improve transparency, each source was evaluated according to its role within the manuscript. Methodological papers were used only to support the reporting structure, whereas clinical and biological sources were used to inform the evidence synthesis and the proposed clinical framework.

Greater weight was assigned to consensus reports, clinical practice guidelines, systematic reviews, meta-analyses and studies with direct relevance to diabetic patients, periodontal inflammation, apical periodontitis, endo-periodontal lesions or HbA1c-related treatment outcomes. Sources with limited clinical applicability, incomplete bibliographic traceability or insufficient relevance to the proposed framework were not used to support key clinical recommendations.

The final evidence base comprised 45 references. Of these, 44 were included in the clinical and biological synthesis: 9 guidelines, consensus reports and position statements; 17 systematic reviews, meta-analyses and umbrella reviews; 11 clinical, interventional and observational studies; and 7 mechanistic studies or contemporary narrative reviews. One additional methodological source was used solely to support the organization and reporting of the review.

### 2.6. Synthesis Strategy

The evidence was synthesized descriptively across five predefined domains: diabetes and periodontal inflammation; diabetes and periapical disease; HbA1c and treatment response; endo-periodontal diagnosis and therapeutic sequencing; and periodontal, surgical or regenerative treatment in metabolically compromised patients. Within each domain, findings were compared according to study design, population characteristics, clinical setting, reported outcomes and direct applicability to dental practice.

Greater interpretive weight was assigned to consensus reports, clinical practice guidelines, systematic reviews and meta-analyses, while observational, interventional and mechanistic studies were used to refine specific diagnostic, prognostic and material-related considerations. Because of the heterogeneity of the available evidence, the synthesis focused on clinically consistent patterns rather than quantitative aggregation.

The proposed framework was developed by integrating these evidence domains into sequential clinical decision points addressing local diagnosis, metabolic risk assessment, treatment priority, timing of invasive procedures and follow-up intensity. The resulting structure was intended to support chairside decision-making while preserving the need for individualized clinical judgment and interdisciplinary medical collaboration when metabolic control is suboptimal.

## 3. Results

### 3.1. Evidence Map

The mapped evidence supports a clinically meaningful relationship between diabetes mellitus, periodontal inflammation and endodontic outcomes. The most substantial and methodologically consistent evidence concerns the bidirectional association between diabetes and periodontitis. Consensus reports, systematic reviews and meta-analyses indicate that poor glycemic control is associated with greater periodontal inflammatory burden and more severe periodontal destruction, while periodontal treatment may contribute to modest short-term reductions in HbA1c, particularly in patients with elevated baseline values.

The evidence concerning endodontic outcomes is comparatively smaller and more heterogeneous, reflecting differences in study design, diabetes definition, glycemic assessment, endodontic diagnosis, treatment quality and follow-up duration. Nevertheless, systematic reviews, meta-analyses and clinical studies consistently suggest that diabetes is associated with a higher prevalence of persistent periapical radiolucency, delayed periapical healing, less favorable outcomes after root canal treatment and increased non-retention of root-filled teeth [18,19,20,21,22,23,24]. These findings support the inclusion of metabolic status in endodontic prognosis and follow-up planning, without treating diabetes as an absolute contraindication to conservative dental treatment.

Evidence related specifically to endo-periodontal lesions remains more limited and is derived mainly from consensus recommendations, retrospective studies, clinical trials and broader periodontal or endodontic literature. The available findings therefore support an integrated interpretation in which pulpal status, periodontal destruction, root integrity, restorability, local infection activity and HbA1c are evaluated together. The clinically relevant evidence domains, representative sources, reported outcomes and their interpretation within the proposed HbA1c-guided framework are summarized in Table 4.

### 3.2. Diabetes, Periodontal Inflammation and Systemic Metabolic Burden

**Figure 2:**
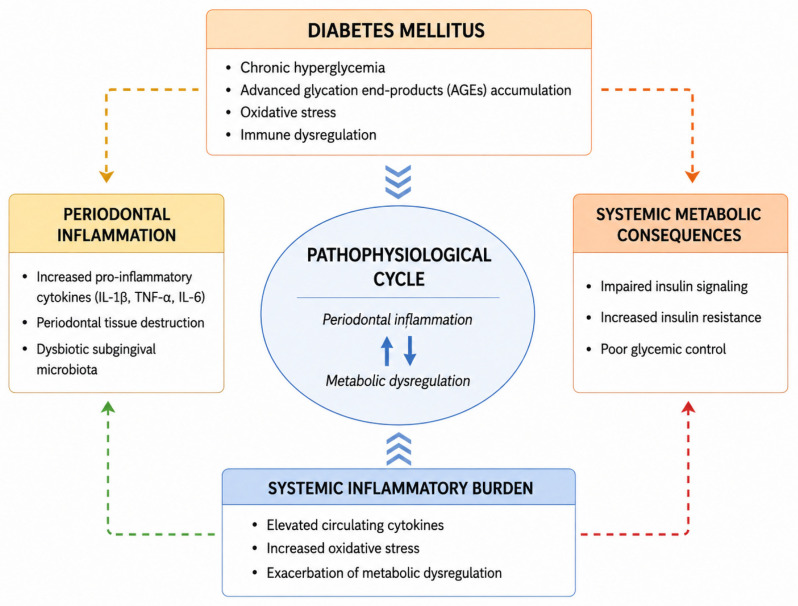
Pathophysiological cycle linking diabetes mellitus, periodontal inflammatory response, systemic inflammatory burden, and metabolic dysregulation. The blue double-headed arrow indicates the bidirectional relationship between periodontal inflammation and metabolic dysregulation.

Diabetes and periodontitis interact through chronic inflammation, dysbiotic biofilms, oxidative stress and impaired immune regulation. Poor glycemic control increases susceptibility to periodontal destruction, while periodontal inflammation increases systemic inflammatory burden and may contribute to insulin resistance [8,9,10,11,12]. These mechanisms explain why patients with poorly controlled diabetes frequently present deeper periodontal pockets, greater clinical attachment loss, higher bleeding tendency and less predictable healing after active periodontal therapy. The bidirectional biological cycle linking diabetes, periodontal inflammation, systemic inflammatory burden and metabolic dysregulation is illustrated in Figure 2.

### 3.3. Diabetes and Periapical Healing

Endodontic prognosis is influenced by several local and systemic factors, including the degree of microbial control, root canal anatomy, the quality of chemomechanical preparation, the initial apical status, the integrity of the coronal seal, the host immune response and the patient’s overall healing capacity. Diabetes may adversely modify this prognosis by impairing neutrophil function, altering microvascular perfusion, sustaining pro-inflammatory cytokine activity and delaying bone remodeling around the periapical lesion [10,11,12,18,19,20,21,22,23,24]. These mechanisms may contribute to slower resolution of apical inflammation and to a less predictable radiographic healing pattern after root canal treatment.

Systematic reviews and meta-analyses support an association between diabetes and a higher prevalence of radiolucent periapical lesions in root-filled teeth. Gupta et al. reported a greater prevalence of periapical lesions among diabetic patients, although pooled estimates varied according to study design and population characteristics [18]. Cabanillas-Balsera et al. identified a higher frequency of non-retention of root-filled teeth in diabetic subjects, with an overall odds ratio of 2.44 [21]. Liu et al., in a meta-analysis of 15 studies, concluded that diabetes increases the risk of apical periodontitis in endodontically treated teeth [22]. Additional clinical and cross-sectional studies further support the relevance of diabetic status in the assessment of apical periodontitis, periapical pathology and post-treatment outcome [23,24].

The available evidence should not be interpreted as indicating that root canal treatment is contraindicated in diabetic patients. Rather, diabetes should be considered a prognostic modifier that may influence the pace and predictability of healing. A recent HbA1c value should therefore be documented whenever possible, particularly in patients with extensive apical lesions, persistent symptoms or a history of delayed healing. The clinician should also explain that radiographic resolution may occur more slowly and that successful treatment remains dependent primarily on adequate infection control, effective irrigation, complete chemomechanical preparation and a durable coronal seal.

In cases in which infection control cannot be achieved predictably in a single visit, intracanal medication may be used to support microbial reduction and control persistent exudate. Follow-up should be adapted to the initial lesion size, clinical symptoms, quality of treatment and metabolic status. Closer clinical and radiographic monitoring may be appropriate for diabetic patients with preoperative apical periodontitis, particularly when glycemic control is suboptimal or when early signs of healing remain uncertain.

### 3.4. Diagnostic Protocol for Diabetic Patients with Suspected Endo-Periodontal Lesions

Diagnosis requires a disciplined sequence. The clinician first determines whether the tooth has pulpal necrosis, irreversible pulpal disease, periodontal breakdown, root damage or a combination of these findings. In diabetes, this local diagnosis must be accompanied by systemic risk documentation because HbA1c influences treatment predictability and recall intensity.

Core clinical steps include medical history, recent HbA1c value, current antidiabetic therapy, presence of cardiovascular or renal comorbidities, smoking status, periodontal charting, tooth mobility, furcation assessment, percussion, palpation, vitality testing, probing pattern and evaluation of restorability. A narrow isolated deep pocket associated with a non-vital tooth may indicate endodontic drainage through the periodontal ligament, whereas generalized deep pockets with bleeding and radiographic horizontal or vertical bone loss support a periodontal component [2,3]. Cracked teeth and vertical root fractures require careful differential diagnosis because they can mimic combined endo-periodontal lesion destruction [31,32].

Periapical radiography remains the first-line imaging method. Cone beam computed tomography (CBCT) should be reserved for complex or atypical cases, suspected root fracture, suspected perforation, resorptive defects, unclear lesion origin, pre-surgical assessment or cases in which two-dimensional imaging cannot explain the clinical pattern [31].

### 3.5. HbA1c as a Prognostic Marker and Planning Variable

HbA1c should be interpreted as a risk modifier rather than an absolute therapeutic threshold. Dental decision-making should also consider acute infection, systemic complications, medication profile, smoking, oral hygiene, periodontal stage, endodontic diagnosis, restorability and patient adherence. Nevertheless, HbA1c categories help clinicians communicate risk and choose an appropriate intervention sequence. In clinical practice, HbA1c values should be used to adjust the intensity, timing and invasiveness of treatment rather than to deny necessary care. Acute pain, swelling, suppuration or spreading infection require prompt endodontic or periodontal infection control, even when glycemic control is poor. In contrast, elective periodontal surgery, regenerative procedures and complex multidisciplinary interventions require a higher level of systemic stability because their success depends on predictable soft-tissue healing, inflammatory resolution and bone remodeling.

The proposed HbA1c categories therefore provide a practical chairside orientation for risk communication and treatment planning. Lower HbA1c values support standard sequencing when local diagnostic criteria are favorable, whereas higher values justify staged therapy, shorter recall intervals, medical communication and postponement of elective invasive procedures. These categories should always be interpreted together with the patient’s general medical status, recent glycemic trend, oral hygiene capacity and the biological severity of the endo-periodontal lesion.

Table 5 summarizes a pragmatic HbA1c-based stratification model for endo-periodontal lesion management. The thresholds are intended to support clinical reasoning, not to function as rigid cut-offs, and each decision should be individualized according to local infection status, periodontal prognosis and medical risk.

## 4. HbA1c-Guided Clinical Practice Framework

### 4.1. General Principles

The proposed framework translates metabolic risk stratification into a practical clinical pathway for patients with endo-periodontal lesions and diabetes mellitus. Its purpose is not to replace local diagnosis, but to organize treatment decisions according to the interaction between pulpal status, periodontal destruction, inflammatory activity and systemic metabolic control. In this context, HbA1c is not interpreted as an isolated therapeutic threshold, but as a systemic risk modifier that may influence healing predictability, treatment timing and the sequencing of endodontic, periodontal and regenerative interventions.

**Figure 3:**
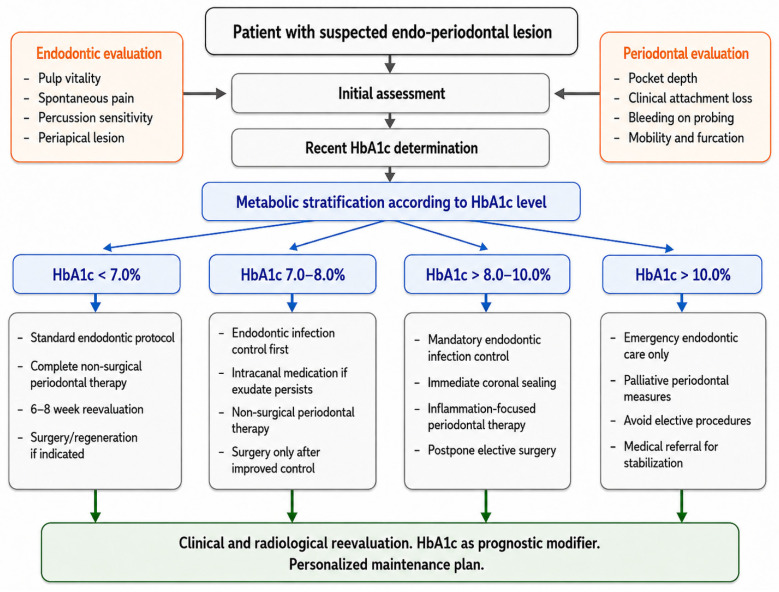
Clinical treatment algorithm for suspected endo-periodontal lesions based on HbA1c values.

Clinical application begins with confirmation of the lesion through periodontal probing, pulp vitality testing, periapical radiography and, when indicated, CBCT assessment. The clinician should first determine whether the lesion is primarily endodontic, primarily periodontal or truly combined, because this distinction directly influences the therapeutic sequence. At the same time, periodontal pocket depth, clinical attachment loss, bleeding on probing, mobility, furcation involvement, periapical status, sinus tract and percussion sensitivity should be assessed in an integrated manner. After the endodontic and periodontal components have been defined, a recent HbA1c value is incorporated into the decision-making process to estimate systemic risk and to guide the intensity and timing of treatment. Figure 3 presents the proposed HbA1c-guided clinical pathway for patients with suspected endo-periodontal lesions.

The first decision point concerns the presence of pulpal necrosis, intracanal infection, sinus tract, suppuration or acute symptoms. In these situations, endodontic infection control has priority, because persistent microbial contamination may maintain periapical inflammation, compromise periodontal healing and contribute to progressive attachment loss. This step should proceed even in patients with poor glycemic control when infection control is clinically necessary. However, in patients with elevated HbA1c, treatment should be planned with greater attention to infection control, coronal sealing, postoperative monitoring and communication with the physician when systemic instability or complications are suspected.

The second decision point concerns the periodontal component. Non-surgical periodontal therapy, plaque control, subgingival debridement and supportive care represent the initial periodontal measures across all HbA1c categories. These interventions reduce inflammatory burden with limited morbidity and create a more stable biological environment for reassessment. In patients with acceptable metabolic control, periodontal treatment may follow standard protocols. In patients with moderate or poor metabolic control, the same principles apply, but the clinician should consider staged therapy, shorter recall intervals, reinforcement of oral hygiene measures and closer monitoring of clinical response.

The third decision point concerns surgical or regenerative therapy. These procedures require greater healing capacity and should be planned only after local inflammation has been controlled, patient adherence has been verified and metabolic status has been reviewed. In patients with elevated HbA1c, elective surgery or regeneration should be postponed whenever possible and coordinated with the physician or diabetologist. This does not mean that dental care should be denied, but rather that invasive elective procedures should be delayed until the biological conditions for predictable healing are improved. Emergency care, drainage, endodontic treatment and palliative periodontal measures remain indicated when clinically necessary.

The HbA1c categories included in the framework provide a practical structure for chairside decision-making. Patients with HbA1c values below 7% may generally be managed through standard endodontic and non-surgical periodontal protocols, followed by reevaluation and surgery or regeneration when indicated. Values between 7% and 8% suggest increased risk and justify prioritization of infection control, careful reassessment and possible postponement of elective surgery until metabolic stability improves. Values above 8.0% and up to 10.0% indicate a higher-risk clinical context in which endodontic infection control and inflammation-focused periodontal therapy should be prioritized, while elective surgical procedures should be approached cautiously. Values above 10.0% suggest severe metabolic imbalance, in which emergency endodontic care, palliative periodontal measures and medical referral for stabilization should take precedence over elective interventions.

Reevaluation represents a mandatory step before definitive periodontal surgery, regenerative therapy or long-term prognosis assignment. Clinical and radiographic reassessment allows the clinician to determine whether residual periodontal defects reflect persistent periodontal destruction, incomplete endodontic healing, poor plaque control or systemic impairment of tissue repair. This stage is particularly important in diabetic patients, because the initial response to therapy may be delayed or less predictable when metabolic control is inadequate. The framework therefore supports staged decision-making rather than a single therapeutic decision made at baseline.

Overall, Figure 3 summarizes this approach by integrating local diagnosis, HbA1c-based metabolic stratification, therapeutic sequencing and maintenance planning in a chairside decision pathway. The figure emphasizes that endodontic and periodontal evaluations should be performed in parallel, followed by recent HbA1c determination and risk-based treatment selection. Regardless of the HbA1c category, all patients require clinical and radiological reevaluation, individualized maintenance planning and interpretation of HbA1c as a prognostic modifier rather than as an absolute contraindication to treatment.

### 4.2. Diagnostic Decision Matrix

Accurate diagnosis of endo-periodontal lesions requires the simultaneous interpretation of pulpal, periodontal, radiographic and systemic findings. In diabetic patients, this process becomes more complex because impaired metabolic control may modify inflammatory expression, delay healing and reduce the predictability of both endodontic and periodontal outcomes.

Therefore, clinical decision-making should not be based on a single parameter, such as pocket depth, pulp vitality or HbA1c alone, but on the combined interpretation of local and systemic indicators.

The diagnostic matrix translates common clinical patterns into practical management priorities. It is intended for chairside use after complete periodontal charting, pulpal testing and radiographic assessment. By correlating pulp findings with periodontal findings and metabolic risk, the matrix helps differentiate primary endodontic lesions with periodontal drainage, primary periodontal lesions, true combined endo-periodontal lesions, vertical root fractures or acute infections in systemically vulnerable patients.

Table 6 summarizes the proposed diagnostic decision matrix and links each clinical pattern to its most likely interpretation and primary management priority.

### 4.3. Therapeutic Sequencing

Therapeutic sequencing in diabetic patients with endo-periodontal lesions should be guided by the origin and activity of infection, symptom intensity, periodontal prognosis, tooth restorability, radiographic morphology, patient adherence and the level of metabolic control.

In this context, HbA1c does not replace local diagnosis, but contributes to the estimation of systemic risk, healing predictability and the appropriate timing of invasive or elective procedures.

The treatment sequence should therefore be individualized rather than applied as a rigid protocol, and the choice of therapeutic materials should reflect both the local clinical findings and the patient’s metabolic status.

When pulpal necrosis, acute apical infection, sinus tract, persistent exudate or spontaneous pain is present, endodontic infection control is prioritized because intracanal microbial persistence can maintain periapical inflammation and communicate with the periodontal compartment through anatomical or pathological pathways. Chemomechanical preparation, appropriate irrigation, control of apical patency when indicated and an effective temporary or definitive coronal seal are essential for reducing the microbial burden and creating conditions for subsequent periodontal healing.

Calcium hydroxide remains a commonly used intracanal medicament when persistent exudate, complex anatomy, extensive periapical involvement or incomplete microbial control prevents predictable treatment in a single visit [33,34]. Its antimicrobial activity, alkaline pH and capacity to contribute to endotoxin neutralization support its use between appointments.

In diabetic patients, however, persistent inflammation, altered immune regulation and reduced tissue-repair capacity may delay clinical resolution [18,19]. Persistent exudate or symptoms should therefore prompt reassessment of irrigation efficacy, working length, missed anatomy, canal permeability and coronal leakage rather than repeated empirical replacement of the same medicament without further diagnostic investigation.

Calcium silicate-based bioceramic materials may be considered during definitive obturation, perforation repair, apical barrier procedures or retrograde filling because of their sealing ability, biocompatibility and bioactive interaction with mineralized tissues. Experimental evidence indicates that selected calcium silicate-based sealers can modulate inflammatory responses and support osteogenic differentiation and mineral deposition [35,36].

These characteristics may be clinically relevant in patients with impaired tissue repair; nevertheless, direct evidence demonstrating superior endodontic outcomes specifically in diabetic patients remains limited. Bioceramic sealers should therefore not be considered substitutes for adequate chemomechanical disinfection or for intracanal medication when the latter is indicated. Their selection should consider canal anatomy, moisture conditions, obturation technique, retreatability, extrusion risk and the quality of the definitive coronal restoration.

The periodontal phase should focus initially on reducing the inflammatory burden through individualized oral hygiene instruction, supragingival and subgingival instrumentation, elimination of plaque-retentive factors and control of acute periodontal inflammation.

These measures are appropriate across all HbA1c categories because they have limited morbidity and can improve the local biological environment before reevaluation. As metabolic control worsens, periodontal treatment should be delivered in staged appointments, accompanied by stronger reinforcement of plaque control, shorter recall intervals and closer monitoring of bleeding on probing, pocket depth and suppuration.

Site-specific local drug-delivery systems may be considered as adjuncts to mechanical periodontal instrumentation in selected residual, recurrent or difficult-to-access periodontal pockets.

Available formulations include chlorhexidine-containing chips, doxycycline or minocycline gels and microspheres, metronidazole gels, fibers, films and other controlled-release systems [37,38,39]. These modalities can achieve high antimicrobial concentrations within the periodontal pocket while limiting systemic exposure. This may be particularly relevant in diabetic patients receiving multiple medications because local delivery limits systemic exposure and may reduce the likelihood of clinically relevant systemic adverse effects. In patients with elevated HbA1c, local delivery may therefore be considered when adjunctive antimicrobial therapy is indicated but systemic administration is undesirable.

Local antimicrobial delivery should not replace adequate subgingival instrumentation, plaque control or correction of local predisposing factors. Its use should be restricted to clearly selected sites and should consider pocket morphology, microbial persistence, previous antimicrobial exposure, allergy history, product availability and the potential for antimicrobial resistance.

Evidence supporting a specific local-delivery agent exclusively for patients with elevated HbA1c remains limited; consequently, the choice of agent should be based primarily on the local periodontal indication rather than on HbA1c alone.

Surgical periodontal therapy, regenerative treatment and complex reconstructive procedures should not be planned at baseline in the presence of uncontrolled local inflammation, inadequate plaque control, poor adherence or metabolic instability. These interventions require predictable vascularization, collagen turnover, bone remodeling and wound stability and should therefore follow reevaluation after endodontic infection control and non-surgical periodontal therapy. When HbA1c indicates poor metabolic control, elective surgery or regeneration should be postponed whenever possible, unless an urgent local indication requires immediate intervention [17,40].

A fixed therapeutic sequence cannot be imposed on all endo-periodontal lesions. The systematic review addressing the timing of periodontal intervention reported very low certainty for universal sequencing recommendations [25]. Retrospective evidence in grade 3 endo-periodontal lesions indicates that periodontal parameters may improve after standardized endodontic treatment, supporting periodontal reassessment before additional intervention [26]. The clinician should therefore combine lesion origin, symptom intensity, restorative prognosis, periodontal stage, radiographic morphology, metabolic status and patient adherence when determining the order and timing of treatment.

The selection of regenerative materials should be adapted to defect morphology, soft-tissue conditions, tooth prognosis and systemic healing capacity. Available approaches include barrier membranes, bone grafts or bone substitutes, enamel matrix derivatives, platelet concentrates and combinations of these materials [27,28,29,30,41,42,43]. In patients with diabetes, hyperglycemia may adversely influence angiogenesis, inflammatory regulation, collagen metabolism and bone formation, thereby reducing the predictability of regenerative therapy.

Periodontal regenerative therapy may be considered in carefully selected patients with type 2 diabetes when local infection is controlled, oral hygiene is adequate, patient adherence is confirmed, and metabolic status is reasonably stable. Enamel matrix derivatives, bone substitutes, barrier membranes, and platelet concentrates may be selected according to defect morphology and the local clinical indication. However, no regenerative material can compensate for persistent inflammation, inadequate plaque control, poor wound stability, or severe metabolic imbalance. In patients with elevated HbA1c, minimally invasive surgical techniques, stable wound closure, and close postoperative monitoring may be preferable, while extensive elective regenerative procedures should be deferred until modifiable systemic and local risk factors have been addressed.

This approach does not deny necessary dental care to patients with poor metabolic control. Emergency drainage, endodontic infection control, management of acute periodontal infection and other low-morbidity measures remain indicated when clinically necessary. Metabolic risk primarily modifies the timing, invasiveness and expected predictability of elective surgical and regenerative procedures.

Because therapeutic sequencing depends on both local infection status and systemic metabolic risk, treatment should be organized into staged clinical phases rather than applied as a single standardized protocol. Each phase should have a clearly defined objective, a specific intervention, an appropriate timing and a measurable reevaluation endpoint. This staged approach allows the clinician to control acute infection, reduce periodontal inflammatory burden, reassess tissue response and determine whether surgical, regenerative or maintenance-oriented therapy is appropriate.

Table 7 summarizes the proposed therapeutic sequencing, material-related considerations and follow-up endpoints for diabetic patients with endo-periodontal lesions.

### 4.4. Regenerative and Surgical Decision-Making

Regenerative procedures may improve periodontal outcomes in carefully selected endo-periodontal lesions, particularly when root damage has been excluded, the tooth is restorable, defect morphology is favorable and adequate endodontic and periodontal infection control has been achieved. Current periodontal guidelines, systematic reviews, network meta-analyses, randomized clinical trials and retrospective clinical studies support selected regenerative approaches, including guided tissue regeneration, bone grafting or bone-substitute materials, platelet concentrates and combined endodontic and periodontal therapy [27,28,29,30,41,42,43]. Adjunctive diode laser therapy has also been investigated in the management of endo-periodontal lesions, although its additional clinical benefit appears to depend on the protocol, lesion characteristics and associated conventional treatment [44].

The decision to proceed with regenerative therapy should be based on a comprehensive assessment of the local and systemic conditions that influence wound healing. Relevant local variables include the depth and configuration of the osseous defect, the number of remaining bony walls, furcation involvement, root anatomy, tooth mobility, soft-tissue quality, plaque control and the possibility of obtaining stable primary closure. The choice between barrier membranes, grafting materials, enamel matrix derivatives, platelet concentrates or combined regenerative approaches should therefore be adapted to defect morphology and surgical feasibility rather than selected according to a uniform protocol.

The diabetic microenvironment may compromise angiogenesis, collagen turnover, bone remodeling, inflammatory resolution and postoperative wound stability, thereby reducing the predictability of membrane-, graft- and platelet-based regenerative procedures [8,9,10,11,12,27,28,29,30,41,42,43]. Regenerative evidence should therefore not be interpreted as guaranteeing a favorable outcome in patients with poorly controlled diabetes. In patients with HbA1c values above 8.0%, priority should generally be given to infection control, non-surgical periodontal therapy, improvement of plaque control, short recall intervals and medical stabilization before elective regenerative procedures are considered. In patients with HbA1c values above 10.0%, elective periodontal surgery should usually be deferred, and interdisciplinary medical evaluation should be encouraged unless urgent dental infection control is required.

A similar risk-oriented approach is relevant to implant-related decision-making in diabetic patients. Metabolic control should be considered together with periodontal stability, smoking, oral hygiene, bone availability and the patient’s ability to comply with long-term maintenance when estimating healing predictability and long-term risk [45]. Elevated HbA1c should not be viewed as an isolated contraindication, but it should influence the timing of intervention, the extent of surgery, postoperative surveillance and risk communication.

Surgical timing also depends on tooth restorability, mobility, furcation grade, strategic value, smoking status and patient adherence. A tooth with advanced attachment loss, vertical root fracture and poor restorability in a patient with poor metabolic control should not be assigned a favorable prognosis solely on the basis of a planned regenerative procedure. In such cases, the expected biological benefit must be weighed against treatment complexity, long-term maintainability and the likelihood of recurrent infection or structural failure.

When surgery is considered appropriate, minimally invasive techniques, atraumatic tissue handling, stable wound closure and close postoperative monitoring may improve procedural predictability. Nevertheless, no regenerative material or surgical technique can compensate for persistent infection, inadequate plaque control, poor adherence or severe metabolic instability. Regenerative treatment should therefore be undertaken only after modifiable local and systemic risk factors have been addressed and the tooth has a realistic long-term maintenance potential.

### 4.5. Data Collection for Future Clinical Validation

The proposed framework requires prospective clinical validation in well-defined patient populations. Future studies should use standardized patient-level variables, including HbA1c, periodontal status, pulpal diagnosis, lesion morphology, treatment sequence, material selection and follow-up outcomes. Consistent data collection would allow evaluation of the framework’s prognostic accuracy and clinical applicability. Retrospective audits may provide preliminary evidence, while prospective cohort studies could assess healing, tooth retention and treatment complications over time. Hypothetical patient data should not be presented as clinical evidence. Table 8 summarizes the recommended variables for future validation studies.

## 5. Discussion

The available evidence supports the clinical relevance of integrating metabolic status into endodontic and periodontal decision-making, but it also shows that HbA1c cannot function as an isolated diagnostic or therapeutic determinant. The strongest data concern the relationship between diabetes and periodontitis, including the effect of periodontal therapy on short-term glycemic markers. Evidence from endodontics is less extensive, but it consistently indicates that diabetes may impair periapical healing and reduce the long-term retention of root-filled teeth. This difference in evidence strength should guide interpretation of the proposed framework.

### Limitations of the Evidence and Framework

This article maps and synthesizes evidence but does not provide a quantitative meta-analysis. The source literature includes heterogeneous study designs, diagnostic definitions, HbA1c thresholds, follow-up intervals and outcome measures. These differences limit direct comparison across studies and prevent calculation of a single pooled estimate for endo-periodontal lesions in diabetic patients.

The proposed HbA1c thresholds are pragmatic and clinically oriented. They derive from diabetic care principles, periodontal guidelines and the observed influence of metabolic control on oral inflammatory healing, but they are not specific validated cut-offs for endo-periodontal lesions. Individual medical context remains essential, especially in older patients, frail patients, patients with renal disease, patients with cardiovascular complications and patients with hypoglycemia risk.

The review was designed as a structured narrative synthesis with an expert-informed clinical framework. Evidence from heterogeneous study types was integrated descriptively to support the proposed clinical decision-making framework. No quantitative pooling was performed because the manuscript was not designed as a systematic review or meta-analysis.

Several endo-periodontal studies have small samples or retrospective designs. Regenerative outcomes are often reported in specialized centers with strict case selection, which may overestimate predictability in routine practice. Future prospective studies should test the proposed algorithm using standardized HbA1c categories, tooth-level diagnosis, follow-up radiography and patient-centered outcomes.

## 6. Future Directions and Clinical Implications

Future research should move from isolated associations toward validated prediction models. Such models should integrate HbA1c, diabetes duration, smoking, plaque control, periodontal stage and grade, vitality status, preoperative apical lesion size, coronal seal quality, furcation involvement and regenerative defect morphology. A tooth-level prediction model would help clinicians communicate prognosis and avoid both overtreatment and premature extraction.

Clinical studies should also assess whether combined periodontal and endodontic stabilization improves systemic markers beyond HbA1c, such as C-reactive protein, IL-6 or other inflammatory mediators. The bidirectional relationship between periodontitis and diabetes suggests that combined oral infection control may contribute to systemic inflammatory reduction, but this hypothesis requires well-designed trials with medical collaboration.

From a practical standpoint, dental clinics should record recent HbA1c in diabetic patients who require complex periodontal, surgical, endodontic or regenerative procedures. A recent value within the previous three months is clinically useful. When values are unavailable or high, the clinician should request medical coordination rather than proceeding automatically with elective invasive therapy.

The framework also supports teaching and interdisciplinary communication. Periodontists, endodontists, general dentists and physicians can use the same risk language when discussing whether the immediate priority is pain control, infection drainage, periodontal debridement, metabolic stabilization, surgery or supportive maintenance.

## 7. Conclusions

Endo-periodontal lesions in diabetic patients result from the interaction of local infection, periodontal inflammation and systemic metabolic dysregulation. Diabetes does not create a distinct pathognomonic lesion, but it changes inflammatory intensity, healing capacity, recurrence risk and long-term prognosis.

HbA1c has practical value in dental decision-making. It helps clinicians stratify risk, plan treatment sequence, determine timing of elective periodontal surgery, intensify maintenance and communicate prognosis. It should complement, not replace, pulpal testing, periodontal charting and radiographic diagnosis.

The proposed HbA1c-guided clinical practice framework prioritizes endodontic infection control when necrosis or active intracanal infection is present, adapts periodontal intervention to metabolic status and reserves elective regenerative therapy for cases with controlled inflammation, favorable local anatomy and acceptable systemic conditions. Prospective validation is required before the framework can be converted into a formal guideline.

## Figures and Tables

**Table 1:** Population–Concept–Context framework used to structure the review question.

PCC Element	Definition Used	Clinical Relevance
Population	Adults with diabetes mellitus or impaired glycemic control, including patients with periodontitis, apical periodontitis, root-filled teeth or suspected endo-periodontal lesions.	Identifies patients in whom systemic metabolic status can modify healing, recurrence and treatment sequencing.
Concept	Endo-periodontal lesions, periodontal inflammation, apical periodontitis, endodontic treatment outcome, tooth retention, HbA1c, periodontal therapy and clinical decision-making.	Connects local infection and inflammation with measurable metabolic risk.
Context	Dental, periodontal and endodontic clinical settings in which clinicians evaluate prognosis, timing of procedures, need for interdisciplinary referral and maintenance planning.	Supports direct translation into daily dental practice.

**Table 2:** Core and targeted search strategy used to guide source identification.

Database	Core Search String	Limits
PubMed	((“diabetes mellitus” OR diabetes OR hyperglycemia OR HbA1c OR “glycated hemoglobin”) AND (periodontitis OR “periodontal disease” OR “periodontal therapy”) AND (“apical periodontitis” OR endodontic OR “root canal” OR “endo-periodontal lesion”)) OR ((“intracanal medicament” OR “calcium hydroxide” OR “calcium silicate” OR bioceramic) AND (endodontic OR “root canal”)) OR ((“local drug delivery” OR chlorhexidine OR doxycycline OR minocycline OR metronidazole) AND (periodontitis OR “periodontal therapy”))	2016–2025; English; reviews, guidelines, consensus reports, clinical or observational studies, and selected mechanistic studies
Scopus	TITLE-ABS-KEY((diabetes OR hyperglycemia OR HbA1c OR “glycated hemoglobin”) AND (periodontitis OR “apical periodontitis” OR endodontic OR “endo-periodontal”)) OR TITLE-ABS-KEY((“intracanal medicament” OR “calcium hydroxide” OR “calcium silicate” OR bioceramic) AND (endodontic OR “root canal”)) OR TITLE-ABS-KEY((“local drug delivery” OR chlorhexidine OR doxycycline OR minocycline OR metronidazole) AND (periodontitis OR “periodontal therapy”))	2016–2025; English; dentistry, medicine, immunology, clinical sciences and biomaterials
Web of Science	TS = ((“diabetes mellitus” OR diabetes OR hyperglycemia OR HbA1c OR “glycated hemoglobin”) AND (periodontitis OR “periodontal disease” OR “periodontal therapy” OR “apical periodontitis” OR endodontic OR “root canal” OR “endo-periodontal lesion”)) OR TS = ((“intracanal medicament” OR “calcium hydroxide” OR “calcium silicate” OR bioceramic) AND (endodontic OR “root canal”)) OR TS = ((“local drug delivery” OR chlorhexidine OR doxycycline OR minocycline OR metronidazole) AND (periodontitis OR “periodontal therapy”))	2016–2025; English; article, review, guideline or selected mechanistic study; full article available

**Table 3:** Eligibility criteria for source selection and evidence mapping.

Criterion	Included	Excluded	Reason
Population	Adults with diabetes, periodontitis, apical periodontitis, root-filled teeth or endo-periodontal lesions.	Animal models, isolated in vitro biofilm studies, pediatric-only populations.	Clinical applicability to routine dental practice was required.
Evidence type	Consensus reports, guidelines, systematic reviews, meta-analyses, RCTs, cohort and retrospective clinical studies, and selected mechanistic studies relevant to contemporary therapeutic materials.	Narrative sources without transparent clinical relevance; isolated case reports in main analysis.	Higher-level and clinically interpretable evidence were prioritized.
Outcomes	HbA1c, periodontal parameters, periapical radiolucency, periapical healing, tooth retention, complications, follow-up outcomes.	Laboratory-only endpoints without relevance to the clinical framework.	Clinically actionable outcomes were prioritized; mechanistic evidence was used only to support material-related interpretation.
Time frame	2016–2025, with foundational classification papers from 2018 included.	Older sources unless essential for classification or methodology.	The article targets recent practice and submission requirements.

**Table 4:** Evidence map of clinically relevant findings used to build the HbA1c-guided framework.

Evidence Domain	Representative Sources	Study Type	Numerical Findings Reported in the Literature	Clinical Interpretation
Periodontitis and diabetes	Sanz et al. 2018 [8]; Wu et al. 2020 [9]; Enteghad et al. 2024 [12]	Consensus, meta-analysis, review	Periodontal therapy associated with HbA1c reduction of approximately 0.27–0.48% at 3 months; T2DM associated with 34% higher risk of periodontitis in cohort data.	Metabolic control and periodontal inflammation should be assessed together.
Periodontal treatment and HbA1c	Simpson et al. 2022 [13]; Chen et al. 2021 [14]; Baeza et al. 2020 [15]; Umezaki et al. 2025 [16]	Cochrane review, meta-analysis, systematic reviews	Cochrane review reported mean HbA1c reduction around 0.43 percentage points at 3–4 months; effect more evident in patients with higher baseline HbA1c.	Non-surgical periodontal therapy is safe and can contribute to short-term metabolic improvement.
Diabetes and periapical lesions	Segura-Egea et al. 2016 [20]; Gupta et al. 2020 [18]; Liu et al. 2023 [22]	Systematic reviews and meta-analyses	Diabetes associated with higher prevalence of radiolucent periapical lesions and increased risk of apical periodontitis in endodontically treated teeth.	Diabetes should modify endodontic prognosis and recall intensity.
Root-filled tooth retention	Cabanillas-Balsera et al. 2019 [21]; Nagendrababu et al. 2020 [19]	Systematic review and umbrella review	Non-retention of root-filled teeth more frequent in diabetic subjects; one meta-analysis reported overall OR 2.44.	Diabetic status should be documented before prognosis assignment.
Endo-periodontal treatment sequencing	Chen et al. 2024 [2]; Friedrich et al. 2023 [25]; Ruetters et al. 2021 [26]	Consensus, systematic review, retrospective study	Evidence favors infection control, individualized sequencing; quality of comparative sequencing evidence remains low.	Endodontic infection control is prioritized when necrosis or active intracanal infection exists.
Regenerative therapy in EPL	Ustaoglu et al. 2020 [27]; AlJasser et al. 2021 [28]; Oh et al. 2019 [29]; Tietmann et al. 2024 [30]	RCTs and retrospective clinical studies	Selected studies report pocket depth reduction, clinical attachment gain and tooth retention up to 89–92% in specialized settings.	Regeneration is case-dependent and should be delayed when metabolic control is poor.

**Table 5:** Practical HbA1c-based clinical stratification for endo-periodontal lesion management. Thresholds are proposed for dental risk orientation and must be individualized medically.

HbA1c Category	Metabolic Interpretation	Expected Clinical Risk	Endodontic Approach	Periodontal Approach
<7.0%	Generally controlled diabetes or near-target control in many adults, depending on medical context.	Healing risk closer to baseline if local infection is controlled and hygiene is adequate.	Perform root canal treatment according to standard protocols; reinforce coronal sealing; usual radiographic review at 6–12 months for apical lesions.	Full non-surgical therapy; surgery or regeneration possible after reevaluation if local criteria are favorable.
7.0–8.0%	Suboptimal but often clinically manageable control.	Moderate risk of delayed soft-tissue and bone healing, especially in advanced periodontitis.	Prioritize disinfection; consider intracanal medication when exudate persists; document prognosis carefully.	Non-surgical periodontal therapy and inflammation control; surgical decisions after a 8–12 week reevaluation and medical communication.
>8.0–10.0%	Poor control or unstable metabolic status in many clinical settings.	Higher risk of delayed healing, infection persistence and reduced regenerative predictability.	Urgent or necessary endodontic infection control should not be delayed; elective complex procedures require caution.	Focus on plaque control, debridement, antimicrobial risk control and short recall; postpone elective surgery until stabilization when possible.
>10.0%	Severe metabolic dysregulation or high-risk status unless medically justified.	Markedly increased risk for postoperative complications and unpredictable wound healing.	Emergency endodontic treatment for pain, swelling or acute infection; coordinate with physician.	Palliative or non-surgical inflammation control only; defer elective periodontal surgery or regeneration and refer for metabolic stabilization.

**Table 6:** Diagnostic decision matrix integrating pulpal, periodontal and metabolic information.

Clinical Pattern	Pulp Findings	Periodontal Findings	Most Likely Interpretation	Primary Management Priority
Deep narrow isolated pocket with sinus tract	Non-vital or necrotic pulp; percussion may be positive.	Localized probing defect; limited generalized periodontitis signs.	Primary endodontic lesion with periodontal drainage.	Root canal disinfection, intracanal medication if exudate persists, coronal seal, reevaluation before periodontal surgery.
Generalized deep pockets with bleeding and vital pulp	Pulp responds within expected limits.	Generalized CAL, BOP, mobility proportional to bone loss.	Primary periodontal lesion.	Periodontal inflammation control and diabetes-oriented maintenance; endodontics only if pulpal signs develop.
Deep pocket, non-vital tooth, vertical bone defect	Necrotic pulp or previous endodontic treatment failure.	Periodontal pocket extends toward apex; possible furcation involvement.	True combined endo-periodontal lesion without confirmed root damage.	Endodontic infection control, non-surgical periodontal therapy, reevaluation, then regenerative/surgical decision if HbA1c allows.
Narrow isolated pocket along root, J-shaped radiolucency	Variable pulp response; may be previously root-filled.	Suppuration or isolated probing on one root surface.	Vertical root fracture or root damage must be excluded.	CBCT or surgical exploration when indicated; avoid regenerative planning until fracture/perforation is ruled out.
Acute pain, swelling, poorly controlled diabetes	Pulp necrosis or acute apical abscess likely.	Periodontal drainage may coexist.	Acute endodontic infection in high systemic risk patient.	Urgent drainage/infection control, medical coordination, defer elective periodontal surgery.

**Table 7:** Therapeutic sequencing, material and pharmacological considerations, and follow-up endpoints for diabetic patients with endo-periodontal lesions.

Stage	Clinical Objective	Intervention and Material Considerations	Timing	Reevaluation Endpoint
Initial visit	Establish diagnosis and systemic risk.	Medical history, recent HbA1c, periodontal charting, pulp testing, periapical radiography; CBCT only if indicated.	Day 0. Treat urgent infection immediately; obtain medical input when metabolic control is poor.	Lesion origin, restorability and systemic risk documented.
Endodontic phase	Control intracanal infection.	Chemomechanical disinfection and coronal seal. Calcium hydroxide if exudate or infection persists; calcium silicate-based bioceramics for obturation or repair when indicated.	Urgent to early. Elevated HbA1c requires closer monitoring but does not delay necessary infection control.	Pain, exudate and sinus tract reduction; secure seal; radiographic baseline.
Periodontal non-surgical phase	Reduce periodontal inflammation.	Oral hygiene instruction and subgingival instrumentation. Local chlorhexidine, doxycycline/minocycline or metronidazole delivery may be used at selected residual sites.	Early or parallel to endodontic care. Use staged visits and shorter recalls when metabolic control is poor.	Reduced BOP and PPD; no suppuration; improved plaque control.
Reevaluation	Assess healing and prognosis.	Repeat periodontal and endodontic assessment; radiographic review; update HbA1c before elective surgery.	6–12 weeks for periodontal response; 6–12 months for periapical healing.	Decision on maintenance, retreatment, extraction, surgery or regeneration.
Surgical/regenerative phase	Treat residual intrabony or furcation defects.	Minimally invasive surgery when feasible. Consider membranes, bone grafts/substitutes, enamel matrix derivatives or platelet concentrates according to defect morphology.	Only after infection control, good plaque control and acceptable metabolic stability; postpone elective regeneration if HbA1c remains high.	CAL gain, PPD reduction, radiographic bone fill and tooth retention.
Maintenance	Prevent recurrence.	Supportive periodontal care, endodontic/radiographic review and glycemic monitoring. Local antimicrobials only for selected recurrent sites.	Every 3–4 months in high-risk patients; otherwise individualized.	Stable BOP/PPD, mobility, radiographic findings and metabolic status.

**Table 8:** Recommended patient-level data collection grid for future clinical validation of the HbA1c-guided framework. The table presents proposed variables for future studies and does not contain original clinical patient data.

Variable Domain	Recommended Variables	Measurement Format	Clinical Purpose
Demographics and systemic status	Age, sex, diabetes type, diabetes duration, antidiabetic therapy, smoking, cardiovascular or renal comorbidity.	Categorical or continuous; anonymized patient ID.	Controls for systemic confounding and risk stratification.
Metabolic markers	HbA1c, fasting glucose when available, date of measurement, physician confirmation.	HbA1c in percent and/or mmol/mol; glucose in mg/dL or mmol/L.	Links healing outcomes with metabolic control.
Periodontal status	PPD, CAL, BOP, plaque score, mobility, furcation, stage and grade.	Tooth-level and patient-level charting.	Quantifies periodontal burden and treatment response.
Endodontic status	Pulp vitality, spontaneous pain, percussion, sinus tract, preoperative AP, root-filled status, coronal seal.	Tooth-level binary and ordinal variables.	Defines lesion origin and endodontic prognosis.
Imaging	Periapical radiography, CBCT indication, lesion size, defect morphology, root fracture/perforation status.	PAI score, mm measurements where feasible, categorical morphology.	Improves differential diagnosis and surgical planning.
Treatment	Endodontic therapy, retreatment, intracanal medication, SRP, surgery, regeneration, antibiotics when indicated.	Date-stamped intervention fields.	Allows sequence-outcome analysis.
Outcomes	Pain resolution, sinus closure, PPD reduction, CAL gain, radiographic healing, tooth retention, complications.	3, 6, 12 and 24-month follow-up.	Validates the HbA1c-guided algorithm.

## Data Availability

No new data were created or analyzed in this study. Data sharing is not applicable to this article.

## Abbreviations

The following abbreviations are used in this manuscript:

AGE	Advanced glycation end product
AP	Apical periodontitis
BOP	Bleeding on probing
CAL	Clinical attachment loss
CBCT	Cone beam computed tomography
EPL	Endo-periodontal lesion
GTR	Guided tissue regeneration
HbA1c	Glycated hemoglobin
IL-1β	Interleukin-1 beta
IL-6	Interleukin-6
OR	Odds ratio
PAI	Periapical index
PCC	Population, concept and context
PPD	Probing pocket depth
RAGE	Receptor for advanced glycation end products
RCT	Randomized controlled trial
SRP	Scaling and root planing
T2DM	Type 2 diabetes mellitus
TNF-α	Tumor necrosis factor alpha
